# Solar drying and organoleptic characteristics of two tropical African fish species using improved low-cost solar driers

**DOI:** 10.1002/fsn3.101

**Published:** 2014-03-07

**Authors:** Moshood K Mustapha, Taiye B Ajibola, Abdulbashir F Salako, Sunmola K Ademola

**Affiliations:** 1 Department of Zoology, University of IlorinIlorin, Nigeria; 2 Department of Physics, University of IlorinIlorin, Nigeria

**Keywords:** *Clarias gariepinus*, moisture, *Oreochromis niloticus*, organoleptic, renewable energy, solar driers, weight

## Abstract

This study was done to evaluate the drying performance, efficiency, and effectiveness of five different types of improved low-cost solar driers in terms of moisture loss from two tropical African fish species *Clarias gariepinus* (African sharp tooth catfish) and *Oreochromis niloticus* (Nile tilapia) and testing the organoleptic characteristics of the dried samples. The driers used were made from plastic, aluminum, glass, glass with black igneous stone, and mosquito net, with traditional direct open-sun drying as a control. A significant (*P* < 0.05) decrease in weight resulting from moisture loss in the two fish species was observed in all the driers, with the highest reduction occurring in the glass drier containing black stone. The rate of weight loss was faster in the first 4 days of drying with black stone-inserted glass drier showing the fastest drying rate with a constant weight in *C. gariepinus* attained on the 11th day and in *O*. *niloticus* on the eighth day. The slowest drier was plastic where a constant weight of the species were recorded on and 13th day and 11th day, respectively. Volunteers were used to assess the organoleptic characteristics of the dried samples and they showed lowest acceptability for the open-sun drying, while samples from the glass drier containing black stone had the highest acceptability in terms of the taste, flavor, appearance, texture, odor, palatability, and shelf-life. The low-cost solar driers were effective found in removing water from the fish resulting in significant loss of weight and moisture. The highest drying time, efficient performance, drying effectiveness, and high acceptability of the organoleptic parameters of the dried products from the black stone-inserted glass drier were due to the ability of the glass and the black stone to retain, transmit, and radiate heat to the fish sample all the time (day and night). These low-cost driers are simple to construct, materials for its construction readily available, easy to maintain and operate, hygienic in use, reliable, effective, occupies less area, dry products faster with increased shelf-life, save man-hour, user-friendly, use renewable energy, protect the drying samples from filthiness, wetness, and invasion by pests, insects, and microbes, with well-dried, high-quality, and better preserved final products. The adoption and use any of these low-cost solar driers by artisanal fishermen and general household in sub-Saharan Africa will not only help in reducing post catch losses, but also ensure food safety and security as there is abundant solar energy in these sub-Saharan African tropical countries for the operation of the driers.

## Introduction

Fish constitutes an important and cheap source of animal protein to millions of people in tropical countries of sub-Saharan Africa. Sustainable utilization of fish as a resource through various means such as processing and preservation will help meet the nutritional, social, economic, and cultural needs of the people in developing countries most especially in sub-Saharan Africa. In spite of fish being a highly nutritious and economically viable food, it is also one of the most perishable because of its suitable medium for growth of microbes after harvest. Spoilage and deterioration is much faster amongst tropical fish species because of high ambient temperature prevalent in the tropics. Most tropical fish species could become unfit for consumption within 12–20 h of capture unless it is subjected to some of processing (Ames et al. 1999) which include preservation by drying. Various factors such as fish health status, parasites, wounds and bruises, mode of capture, handling, and preservation after capture are responsible for fish spoilage (Akinneye et al. 2007; Tawari and Abowei 2011). Chemical, microbial, and enzymatic actions on captured fish bring about spoilage with resultant deterioration in the flesh, body tissues, and organs of the fish through physical and biochemical changes (Ghaly et al. 2010).

The declining catches of fish from the wild and underdevelopment of fish aquaculture in sub-Saharan Africa make fish preservation from the available ones gotten from the two systems to be imperative. Various traditional methods of fish preservation are carried out in sub-Saharan Africa. These include air drying and smoking by the use of heat in the smoking kilns, salting, or brining involving the use of common salt (sodium chloride), sun drying through exposure to direct sunlight, solar drying where fish is dried in an enclosure which traps sunlight energy. Drying of fish increases the shelf-life, maintains the quality of the fish in terms of its nutrient, flavor, texture, and appearance, provides ease of handling and further processing of the fish, and reduces post catch losses thereby ensuring continuous availability of cheap animal protein to the people all year round.

The development of solar driers has significantly improved the traditional preservation of fish by sundry in developing countries of sub-Saharan African. The driers provide hygienic conditions for fish drying and they could be constructed by simple technology from inexpensive and readily available materials. They can also be easily operated with effective and efficient performance. The effectiveness, efficiency, and performance of various solar driers for drying fish have been described (Malviya and Gupta 1985; Osei-Opare and Kukah 1988; Bala and Mondol 2001; Mazid and Kamal 2005; Sengar et al. 2009; Oparaku 2010; Oparaku et al. 2010; Hii et al. 2012; Rahman et al. 2012).

Along with the evaluation of the effectiveness, efficiency, and performance of the low-cost solar driers in terms of moisture reduction, it is also desirable to analyze the organoleptic characteristics of the final dried products coming from the low-cost solar driers. This is usually done by means of human senses or organs to test for some properties on the dry product. Rahman et al. (2012) used appearance, flavor, texture, filthiness, scales, wetness, and saltiness as organoleptic characteristics of dried fish products. Several studies have been done on the organoleptic characteristics of various fish species dried by different methods. These include Reza et al. (2009), Ojutiku et al. (2009), Effiong and Fakunle (2012), Oparaku and Mgbenka (2012), Rahman et al. (2012), Ogbonnaya and Ibrahim (2009), Oparaku (2010) Oparaku et al. (2010), Tao and Linchun (2008), Huda et al. (2010) among others.

The objective of this study was to dry two commercially important tropical African fish species *Clarias gariepinus* (African sharp tooth catfish) and *Oreochromis niloticus* (Nile tilapia) using different improved low-cost solar driers. This is with the aims of evaluating the performance, efficiency, and effectiveness of these improved low-cost solar driers in terms of moisture loss from the fish samples and testing the organoleptic characteristics of the dried products.

## Materials and Methods

Five different solar driers with a square size of square size of 2 × 2 ft. Inside the driers were placed a wooden stand having a dimension of 1.5 × 1.5 × 0.5 ft (length, width, and height), and a 1.7 × 1.7 ft wire mesh in which the fish species were placed was put on top of the stand. The solar driers were constructed from inexpensive and readily available materials were used for drying of the two fish species. The driers used were as follows:

1. Plastic drier: This was constructed using a thermopile plastic material.
2. Mosquito net drier: This was constructed by using plywood for the frame (edges). The drier was subsequently covered with mosquito net all around the wooden frame.
3. Glass drier: This was made of transparent glass.
4. Aluminum drier: This was constructed from aluminum sheet. The drier was, however, coated both inside and outside with black paint.
5. Glass drier containing black stones: This is similar to the glass drier in every respect but with a black (igneous rock) stone placed in it.

A control which was the direct open-sun drying was set up. The fish species were placed on a 2 × 2 ft steel plate and exposed directly to the sun. The control was not enclosed. All the driers including control were placed at the top of a one-storeyed building at the Physics laboratory of the University of Ilorin, Ilorin, Nigeria with no obstruction to sun rays and facing the direction of the prevailing mind.

### Sample treatment

A total of 48 fresh fish samples of *C. gariepinus* (*n* = 32) and *O. niloticus* (*n* = 16) used for this work were obtained from Kwara State Ministry of Agriculture, Ilorin, Nigeria. The fish samples were weighed and the total length measured. They were descaled (*O. niloticus* only) gutted and mob-dried. The fish samples were then reweighed and measured before placing them in each drier and the control. The experiment was replicated twice.

### Measurement of moisture and weight losses

Moisture and ultimately weight losses from each fish sample and drier were measured daily using a mettler balance until a final constant weight and moisture loss were obtained. The percentage moisture content from each drier for the fish samples was calculated according to Ranganna (1986) as follows:


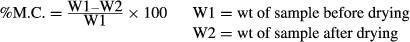


### Measurement of the driers temperature

Maximum and minimum temperatures in the solar driers were measured using a laser sensor thermometer.

### Assessment of organoleptic properties of dried samples

Some organoleptic properties such as appearance, texture, palatability, flavor, odor, shelf-life, and general acceptability of the dried samples *C. gariepinus* which is a fatty fish and *O. niloticus* which is a lean fish from the different driers were evaluated by 10 randomly chosen adult volunteers (age > 20) comprising of five males and five females according to the method of Poste et al. (1991). Qualitative descriptive analysis (QDA) of the organoleptic properties of the dried fish samples was used in evaluating the sensory qualities after taking the samples directly from the driers. The volunteers were asked to judge the organoleptic properties of the dried samples using a 5-point hedonic scale which were as follows: very good (5), good (4), fair (3), poor (2), and bad (1). The shelf-life of the products by volunteers' assessment was evaluated according to New Zealand Food Safety Authority (2005) and Chavan et al. (2011). The evaluations by the volunteers were done in two replicates.

### Evaluation of the performance, efficiency, and effectiveness of the driers

The performance, efficiency, and effectiveness of the solar driers were evaluated through the materials used for their construction, maintenance and operation, time of drying the samples, moisture contents, and organoleptic assessment of the final dried samples.

### Statistical Analysis

Statistical Analysis of the results was done using SPSS (Chicago, IL) (15) using analysis of variance and was further classified by multiple compare tests. Differences between the mean values among the driers, days of drying, and the fish species were determined by Least Significant Difference and Duncan's New Multiple Range Test with significance at *P* < 0.05.

## Results

**Figure 1:**
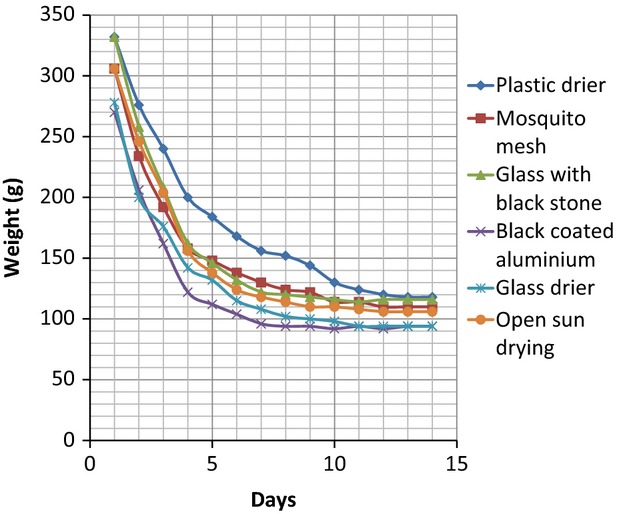
Variations in weight loss (moisture reduction) with time in *Clarias gariepinus* under different improved low-cost solar driers and the open-sun drying.

**Figure 2:**
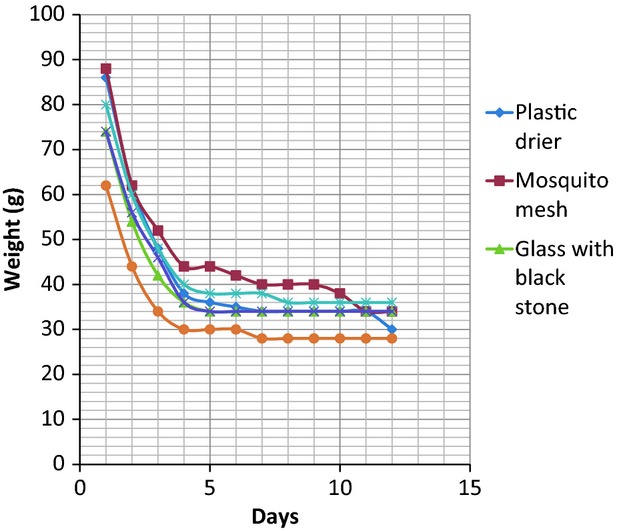
Variations in weight loss (moisture reduction) with time in *Oreochromis niloticus* under different improved low-cost solar driers and the open-sun drying.

The results of the drying (weight and percentage moisture content losses and the final percentage moisture content) of the two fish samples *C. gariepinus* and *O. niloticus* from the different solar driers and the control are presented in Tables 1, 2. The mean weight of *C*. *gariepinus* used for the drying was 336 ± 30 g, while that of *O. niloticus* was 110 ± 20 g. A significant (*P* < 0.05) decrease in weight resulting from moisture loss in the two fish species samples was observed in all the driers, with the highest reduction occurring in the glass drier containing black stone. The variations in weight loss resulting from moisture reduction with time in the two species are shown in Figures 1, 2. A progressive decrease in weight of *C*. *gariepinus* was recorded from the first day of drying until a constant weight was recorded on the 13th day (Fig. 1). The rate of weight loss was faster in the first 4 days of drying, but slowed down afterwards till a constant weight was recorded. Black stone-inserted Glass drier showed the fastest drying rate with a constant weight in *C. gariepinus* attained on the 11th day, while slowest drier was plastic where a constant weight of the species was recorded on and 13th day. The rate of drying was also faster in the first 4 days in all the driers for *O*. *niloticus* shown by the progressive decrease in weight recorded in the species (Fig. 2). A constant weight of *O*. *niloticus* was observed on the eighth day in the black stone-inserted drier, which was the fastest while the plastic drier showed the lowest drying time with a constant weight of *O*. *niloticus* achieved on the 11th day.

**Table 1:** Weight and percentage moisture losses of *Clarias gariepinus* in the solar driers and open-sun drying

Driers	Weight before gutting (g)	Weight after gutting (g)	Weight of gutted sample after drying (g)	Weight loss of gutted dried sample (g)	Percentage moisture loss	Percentage moisture content
Plastic	424	332	118	214	64.45	11.2
Mosquito net	436	374	124	250	66.84	11.02
Glass	392	306	194	212	69.28	10.82
Aluminum	386	304	92	212	69.73	10.95
Glass with black stone	466	374	116	258	68.98	10.77
Open-sun drying (control)	402	346	106	240	69.36	10.84

**Table 2:** Weight and percentage moisture losses of *Oreochromis niloticus* in the solar driers and open-sun drying

Driers	Weight before gutting (g)	Weight after gutting (g)	Weight of gutted sample after drying (g)	Weight loss of gutted dried sample (g)	Percentage moisture loss	Percentage moisture content
Plastic	181	118	30	88	74.57	3.99
Mosquito net	171	122	34	88	72.13	3.68
Glass	152	104	38	66	63.46	3.95
Aluminum	175	120	34	86	71.66	3.79
Glass with black stone	150	126	40	86	68.25	3.6
Open-sun drying (control)	153	92	28	64	69.56	3.75

Temperature readings from the five driers showed that black stone inserted glass drier had the highest maximum and minimum temperatures, while mosquito net drier had the lowest maximum and minimum temperatures. The control showed the lowest maximum and minimum temperatures among all the treatments (Table 3).

**Table 3:** Mean maximum and minimum temperatures in the solar driers and open-sun drying

Driers	Maximum temperature °C	Minimum temperature °C
Plastic	50.00	22.00
Mosquito net	46.00	21.00
Glass	56.5	20.50
Aluminum	59.00	24.00
Glass with black stone	60.5	24.50
Open-sun drying (control)	38.00	20.00

Organoleptic assessment by the volunteers who evaluated the dried samples from the driers and the control revealed that the control had lowest acceptability, while samples from the glass drier containing black stone had a very high acceptability in terms of the taste, flavor, appearance, texture, odor, palatability, and shelf-life as shown in Table 4.

**Table 4:** Qualitative evaluation of some organoleptic characteristics of the dried fish samples from the different solar driers and open-sun drying

Driers	Appearance	Odor	Taste	Flavor	Texture	Palability	Acceptability	Shelf-life (1–30 days)
Plastic	Attractive	Pleasant	Fair	Fair	Hard	Good	Moderate	Long
Mosquito net	Attractive	Pleasant	Good	Good	Very hard	Good	High	Long
Glass	Very attractive	Pleasant	Very good	Very good	Very hard	Good	Very high	Longer
Aluminum	Attractive	Pleasant	Good	Good	Very hard	Good	High	Longer
Glass with black stone	Very attractive	Very pleasant	Very good	Very good	Very hard	Very good	Very high	Longest
Open-sun drying (control)	Not attractive	Not pleasant	Fair	Fair	Hard	Fair	Low	Not long

## Discussion

Sun drying of fish is one of the traditional ways of preserving fish in many sub-Saharan African countries due to the abundance of high solar radiance for most part of the year. Sun drying removes water from the fish by evaporation (Eyo 2001) and this consequently slow down or stop autolytic activity, enzymatic reactions, and microbial activities. Eyo (2001) also reported that high moisture content does not only encourage bacterial and biochemical reactions that can lead to spoilage, but also open way for pest invasion.

Sun drying of fish is usually done in the open where the fish is at the mercy of the weather and is exposed to contamination by dust, insect, pest, bird, and animal attack, infection by microorganisms, enzymatic reaction leading to poor quality and spoilage of preserved fish. In order to mitigate these challenges, solar driers of different models have been developed (Oparaku 2010; Bala and Hossain 2012; Rahman et al. 2012). Solar driers speed up drying process significantly resulting in high-quality product with extended shelf-life.

The five different solar driers used in this work were found to dry the two fish samples *C*. *gariepius* and *O*. *niloticus* effectively by removing water (dehydration) from the fish resulting in significant loss of weight and moisture. The percentage moisture loss was between 64.45 and 69.73 which were very significant considering that fact that a fish with percentage moisture loss of between 66 and 75 will not be infected by microbes and the shelf-life of the fish will be increased (Clucas 1982; Frazier and Westhoff 1998). Complete drying to constant weight was achieved in the least drier (Plastic) on the 13th day for *C. gariepinus* and 11th day for *O. niloticus*. The reason for this was due to the inability of the plastic drier to effectively absorb and concentrate the solar radiation to efficiently dry the fish samples. This could be seen in the mean maximum and minimum temperatures recorded in the drier. The most effective and efficient drier that gave the best performance in terms of complete drying, during time, and solar absorbance was the glass drier containing the igneous rock black stone. In this drier, drying of *C. gariepinus* and *O. niloticus* was achieved on the 11th and eighth days, respectively. The weight loss (moisture reduction) after drying was highly significant in this drier compared to others with percentage moisture loss of 68.98 and 68.25 recorded for *C. gariepinus* and *O. niloticus,* respectively. The highest drying time, efficient performance, and drying effectiveness of the black stone-inserted glass drier were due to the ability of the glass and the black stone to retain, transmit, and radiate heat to the fish sample all the time (day and night). This is evident from the high mean maximum and minimum temperatures recorded in the drier. Hollywood et al. (1996) has also used black-painted rocks as solar collectors to release heat at night in his hybrid solar drier. Black color is known to promote solar absorption. Mosquito net drier also produced excellent moisture losses from the fish samples but the short comings of the drier include its inability to effectively retain the absorbed solar energy and radiate in to the fish samples as the heat tends to diffuse out of the net. The mean maximum and minimum temperatures recorded in the drier lend credence to this fact. Other drawbacks of the mosquito net drier include tearing and porosity of the net which makes it susceptible to invasion by microbes and intrusion by liquid such as rain.

The control (open-sun drying) also produced a good drying for the fish samples with a high-percentage moisture loss. However, because the drying was not done under an enclosure, the fish were invaded and infested by insects, pests, and bacteria. Maggots laid by houseflies were observed on the first day of the drying in the control. This occurred as a result of the moisture present on the fish. Eyo (2001) have reported this scenario in open-sun drying of fish.

Qualitative evaluation of some organoleptic properties of the dried fish samples assessed by means of sense organs of the volunteers showed that fish samples from black stone-inserted glass drier had the highest acceptability. The taste, flavor, odor, appearance texture, shelf-life, and palatability were very good. Dried fish samples from the control was least accepted, because, its organoleptic parameters tested were not so good. This observation agrees with the sensory evaluation done on fish dried by open-sun drying and low-cost solar driers by Sengar et al. (2009) and Rahman et al. (2012). Thus, drying in an enclosure is more effective and has more acceptability in terms of organoleptic properties and increased shelf-life than open-sun drying as reported by Bhandary (1988) and Chavan et al. (2011). The volunteers' assessments could be related to the organoleptic properties of dried samples, while the long shelf-life was due to low moisture content of the species after drying.

## Conclusion

The five solar driers used in this work especially the black stone-inserted glass drier were all found to be effective, efficient, and performed very well in drying the fish samples better than the traditional open-sun drying method. These driers also compared well with other low-cost driers developed and in use in some developing countries such as India, Bangladesh, Philippines, Zimbabwe, and Ghana. These driers are simple to construct, materials for its construction readily available, cheaper in terms of cost of materials and construction. They are easy to maintain and operate, hygienic in use, reliable, effective, occupies less area, dry products faster with increased shelf-life, save man-hour, user friendly, use renewable energy, protect the drying samples from filthiness, wetness, and invasion by pests, insects, and microbes. These driers produced better-preserved, high-quality, well-dried final products. The adoption and use any of these low-cost solar driers by artisanal fishermen and general household in sub-Saharan Africa will not only help in reducing post catch losses, but also ensure food safety and security as there is abundant solar energy in these sub-Saharan African tropical countries for the operation of the driers.

## References

[b1] Akinneye JO, Amoo IA, Arannilewa ST (2007). Effect of drying methods on the nutritional composition of three species of (*Bonga* sp., *Sardinella* sp. and *Heterotis niloticus*. J. Fish. Int.

[b2] Ames G, Clucas I, Paul SS (1999). Post-harvest losses of fish in the tropics.

[b3] Bala BK, Hossain MA, Hii CL, Ong SP, Jangam SV, Mujumdar AS (2012). Solar drying of fishery products. Solar drying: fundamentals, applications and innovations.

[b4] Bala BK, Mondol MRA (2001). Experimental investigation on solar drying of fish using solar tunnel dryer. Drying Technol.

[b5] Bhandary CS (1988).

[b6] Chavan BR, Yakupitiyage A, Kumar S (2011). Drying performance, quality characteristics, and financial evaluation of Indian Mackerel (*Rastrilliger kangurta*) dried by a solar tunnel dryer. Thammasat Int. J. Sci. Tech.

[b7] Clucas JJ (1982). Fish handling processing and preservation in the tropics. Part 2.

[b8] Effiong BN, Fakunle JO (2012). Proximate and mineral content of traditional smoked fish species from Lake Kainji, Nigeria. Bull. Environ. Pharmacol. Life Sci.

[b9] Eyo AA (2001). Fish processing technology in the tropics.

[b10] Frazier WC, Westhoff DC (1998). Food microbiology.

[b11] Ghaly AE, Dave D, Budge S, Brooks MS (2010). Fish spoilage mechanisms and preservation techniques: review. Am. J. Appl. Sci.

[b12] Hii CL, Ong SP, Jangam SV, Mujumdar AS (2012). Solar drying: fundamentals, applications and innovations.

[b13] Hollywood N, Brown S, Toreu B (1996). A design for improved efficiency in solar drying of cocoa. Cocoa Grow. Bull.

[b14] Huda N, Deiri RS, Ahmed R (2010). Proximate, color and amino acid profile of Indonesians traditional smoked catfish. J. Fish. Aquat. Sci.

[b15] Malviya MK, Gupta RS (1985). Design and development of a natural convection solar dryer. J. Agric. Eng.

[b16] Mazid MA, Kamal M (2005). Development of low cost solar dryer for the production of improved quality dried fish.

[b17] New Zealand Food Safety Authority (2005). A guide to calculating the shelf life of foods.

[b18] Ogbonnaya C, Ibrahim MS (2009). Effects of drying methods on proximate compositions of catfish (*Clarias gariepinus*. World J. Agric. Sci.

[b19] Ojutiku RO, Kolo RJ, Mohammed ML (2009). Comparative study of sun drying and solar tent drying of *Hyperopisus bebe occidentalis*. Pak. J. Nutr.

[b20] Oparaku NF (2010). Comparative study of sun and solar cabinet fish dryer of three freshwater fish. Cont. J. Fish. Aquat. Sci.

[b21] Oparaku NF, Mgbenka BO (2012). Effects of electric oven and solar dryer on a proximate and water activity of *Clarias gariepinus* fish. Eur. J. Sci. Res.

[b22] Oparaku NF, Mgbenka BO, Eyo JE (2010). Proximate and organoleptic characteristics of sun and solar dried fish. J. Anim. Res. Int.

[b23] Osei-Opare F, Kukah A (1988).

[b24] Poste LM, Mackie DA, Butler G (1991). Laboratory methods for sensory analysis of food.

[b25] Rahman MJ, Karim E, Shahab Uddin M, Zaher M, Haque MA (2012). Development of low-cost emergency fish dryer in Bangladesh to use in absence of sunlight. Bangladesh Res. Publ. J.

[b26] Ranganna S (1986). Manual of analysis of fruit and vegetable products.

[b27] Reza MS, Bapary MA, Islam MN, Kamal M (2009). Optimization of marine fish drying using solar tunnel dryer. J. Food Process. Preserv.

[b28] Sengar SH, Khandetod YP, Mohod AG (2009). Low cost solar dryer for fish. Afr. J. Environ. Sci. Technol.

[b29] Tao W, Linchun M (2008). Influences of hot air drying and microwave drying on nutritional and odorus properties of grass carp (*Ctenophargodon idellus*) fillets. Food Chem.

[b30] Tawari CC, Abowei JFN (2011). Traditional fish handling and preservation in Nigeria Asian. J. Agric. Sci.

